# The Genetic Polymorphisms of NLRP3 Inflammasome Associated with T Helper Cells in Patients with Multiple Myeloma

**DOI:** 10.1155/2018/7569809

**Published:** 2018-08-23

**Authors:** Xueyun Zhao, Mingqiang Hua, Shuxin Yan, Jie Yu, Fengjiao Han, Chaoqin Zhong, Ruiqing Wang, Chen Zhang, Ming Hou, Daoxin Ma

**Affiliations:** ^1^Department of Hematology, Qilu Hospital of Shandong University, Jinan, Shandong 250012, China; ^2^Department of Hematology, Baotou Central Hospital, Baotou, Inner Mongolia Autonomous Region 014040, China

## Abstract

The pathogenesis of multiple myeloma (MM) remains unclear and the NLRP3 inflammasome has been more and more recognized in the progression of many diseases. To investigate the role of the NLRP3 inflammasome in MM, we determined the genetic polymorphisms and expression of NLRP3 inflammasome-related genes (IL-1*β*, IL-18, CARD8, and NF-*κ*B) in MM patients, and explored their clinical relevance. Furthermore, we investigated the relationship of the NLRP3 inflammasome with Th cells in MM. Our study showed that the CARD8-C10X (rs2043211) AT genotype contributed to the susceptibility of MM. CARD8-C10X TT patients had earlier clinical stage. The WBC count in the three CARD8 genotypes showed an increasing trend (AA<AT<TT). Compared with patients with NF-*κ*B-94 ins/del ATTG ins/ins and ins/del, patients with del/del had the highest myeloma cell ratio. Patients with IL-18 (rs16944) TT had the highest hemoglobin concentration (GG<GT<TT). Furthermore, we found that the genotype of CARD8-C10X (rs2043211) or NF-*κ*B-94 ins/del ATTG was closely related to the frequency of Th1. Therefore, the genetic polymorphisms of the NLRP3 inflammasome associated with Th cells might be involved in the pathogenesis of multiple myeloma.

## 1. Introduction

Multiple myeloma (MM) is a life-threatening plasma cell malignancy, for which the pathogenesis is unclear and standard therapy is inadequate. Many genetic and immunological alterations are involved in the pathogenesis of MM. It is of vital importance to explore its immunological mechanism and find the therapeutic target for MM.

Inflammasomes play key roles in the immune system, which can trigger innate immune defenses and promote adaptive immune responses [1]. Some studies have shown that inflammasomes are involved in the pathophysiology of MM [2]. The most well-known inflammasome is NLRP3. It is a protein complex, including NLRP3 (NLR family, pyrin domain-containing 3), the adaptor ASC (apoptosis-associated speck-like protein containing a C-terminal caspase-recruitment domain), and caspase-1, which is responsible for the maturation and secretion of proinflammatory cytokines, such as interleukin IL-1*β* and IL-18 [3]. Moreover, the caspase-recruitment domain-containing protein 8 (CARD8) controls the activity of caspase-1 and regulation of caspase-1-mediated IL-1 and IL-18 activation [4]. NF-*κ*B, another key molecule of inflammasome, is involved in the growth and survival of myeloma cells in the bone marrow microenvironment. IL-6 is also important for the growth and survival of myeloma cells, and it can stimulate osteoclastogenesis. The correlation between IL-6 and inflammasomes is rarely reported. One of the functions of the NLRP3 inflammasome is the regulation of Th1 and Th17 responses by affecting the secretion of its effector molecules that can markedly affect T cell-mediated autoimmunity [5]. In addition, a published report has shown that NLRP3 is expressed during the differentiation of CD4+ T cells and is specifically involved in the polarization of Th2 cells [6]. These *in vitro* and *in vivo* studies suggest that the NLRP3 inflammasome is closely related to T helper cells, and they interact with each other to participate in the onset and progression of inflammatory, immune, or neoplastic diseases, such as pulmonary inflammation, multiple sclerosis, and lung cancer [5, 6]. The detailed relationship of the NLRP3 inflammasome and Th cells in MM has not been clarified until now.

Genetic polymorphisms involved with the NLRP3 inflammasome were linked to various diseases, such as rheumatoid arthritis, ankylosing spondylitis, melanoma, liver cancer, lung cancer, and ovarian cancer [7–9]. Meanwhile, a truncating polymorphism (rs2043211) can produce a nonfunctional CARD8 and induce an amplification of the inflammatory process, or even evolve into cancer [4]. However, the function of the NLRP3 inflammasome in MM has not yet been clearly clarified. To illuminate the roles of the NLRP3 inflammasome in MM, we determined the polymorphism and expression of NLRP3 inflammasome-related genes in MM patients. Moreover, we investigated their relationship with Th cells and explored their effects in the pathogenesis of MM.

## 2. Materials and Methods

### 2.1. Patients and Controls

A total of 355 MM patients (138 females and 217 males) with a median age of 60 (25–89) years and 350 controls (148 females and 202 males) with a median age of 55 (26–90) years were included in the study for the detection of genetic polymorphisms (Table 1). 42 newly diagnosed MM patients were recruited for testing the genetic polymorphisms and the mRNA expressions of NLRP3 inflammasome-related genes and the plasma levels of effector molecules IL-1*β*, IL-18, and IL-6 in BM. Furthermore, 24 newly diagnosed MM patients were recruited for analyzing the genetic polymorphisms of NLRP3 inflammasome-related genes and the percentage of T helper cell subsets (Th1/Th2/Th9/Th17/Th22/Treg) in peripheral blood. The patients were enrolled from Qilu Hospital, Shandong University between April 2007 and November 2017. The diagnosis of multiple myeloma was based on the International Myeloma Working Group criteria [10]. The Durie-Salmon (DS) clinical staging system and International Staging System (ISS) were used in staging the subjects with MM.

This study was approved by the Medical Ethical Committee of Qilu Hospital, Shandong University, China. Informed consent was obtained from all patients before enrollment in the study in accordance with the Declaration of Helsinki.

### 2.2. DNA Extraction and Genotyping

DNA was extracted from bone marrow or peripheral blood samples of 355 MM patients and 350 controls following the manufacturer's instruction of the TIANamp blood DNA kit (Tiangen Biotech, Beijing, China). The detection reagent of the three SNPs, IL-1*β* (rs16944), IL-18 (rs1946518), and CARD8-C10X (rs2043211), were purchased from Thermo Fisher Scientific (Cat. # 4351379). NF-*κ*B-94 ins/del ATTG promoter polymorphism was detected using the forward primer: 5′-CCG TGC CTG CGT T-3′, reverse primer: 5′-GCT GGA GCC GGT AGG GAA-3′ as well as probe 1: 5′-VIC-ACC ATT GAT TGG GCC-MGB-3′ and probe 2: 5′-FAM-CGA CCA TTG GGC C-MGB-3′ (Thermo Fisher Scientific, USA). All PCR reactions contained 1 *μ*L of DNA, 0.15 *μ*L of TaqMan Universal PCR Master Mix, 1.85 *μ*L of ddH_2_O, and 3 *μ*L of Allelic Discrimination Mix. Real-time PCR was performed on an ABI 7500 Real-Time PCR System (SDS, PE Biosystems) using the following conditions: 50°C for 2 min, 95°C for 10 min, and then 40 cycles of amplification (92°C denaturation for 15 s, 62°C annealing/extension for 60 s). Genotypes were analyzed using the ABI 7500 Sequence Detection System (SDS).

### 2.3. RNA Extraction and Quantitative Real-Time PCR

Bone marrow mononuclear cells (BMMCs) or peripheral blood mononuclear cells (PBMCs) were isolated by Ficoll density gradient centrifugation. Total RNA was extracted from BMMCs of 42 MM patients and PBMCs of 24 MM patients using Trizol (Invitrogen, USA) according to the manufacturer's instruction. Approximately 1 *μ*L of total RNA was reversely transcribed into first-strand cDNA in a final volume of 20 *μ*L using PrimeScript RT Reagent Kit Perfect Real Time (Takara Bio). Reverse transcription reaction was done at 37°C for 15 min, followed by 85°C for 5 s. Real-time PCR was conducted using the LightCycler 480 II Real-Time PCR System (Roche Life Science, Switzerland) following the manufacturer's protocols. The real-time PCR contained, in a final volume of 10 *μ*L, 5 *μ*L of 2 × SYBR Green Real-Time PCR Master Mix, 1 *μ*L of cDNA, 0.8 *μ*L of the forward and reverse primers, and 3.2 *μ*L of ddH_2_O. PCR primers of related genes were shown in Table 2. All experiments were conducted in triplicate. The PCR products were analyzed by melt curve analysis and agarose gel electrophoresis to determine product size and to confirm that no by-product was formed. The results were expressed relative to the number of GAPDH transcripts used as an internal control.

### 2.4. Enzyme-Linked Immunosorbent Assay (ELISA) for IL-1*β* and IL-18

The bone marrow supernatants from 42 MM patients were obtained by centrifugation and stored at −80°C for determination of cytokines. The concentrations of IL-1*β* (Cat. # BMS-224-2, eBioscience, San Diego, USA), IL-18 (Cat. # 85-BMS267-2, eBioscience, San Diego, USA), and IL-6 (Cat. # LH-E10103HU, Wuhan Liuhe Biological Technology Co., Ltd., China) were determined by the ELISA method in accordance with the manufacturer's recommendations. The concentrations were calculated from a standard curve according to the manufacturer's protocol.

### 2.5. Flow Cytometric Analysis of Th1/Th2/Th9/Th17/Th22/Treg Cells

Intracellular cytokines were studied by flow cytometry to trigger the reflex of the cytokine-producing Th cells for 24 MM patients. Briefly, heparinized whole blood (100 *μ*L) with an equal volume of Roswell Park Memorial Institute- (RPMI-) 1640 medium was incubated for 4 h at 37°C in 5% CO_2_ in the presence of 2.5 ng/mL of phorbol myristate acetate (PMA), 1 mg/mL of ionomycin, and 1.7 mg/mL of monensin (all from Alexis Biochemicals, San Diego, CA, USA). PMA and ionomycin were enough to induce many kinds of living cells to secrete cytokines. The mixture can be used to stimulate the cytokine production from *in vitro* cultured cells. Monensin is used to block the intracellular transport mechanisms, thereby leading to an accumulation of cytokines in the cells. After incubation, the cells were stained with 5 *μ*L of Anti-Human CD3 PE-Cyanine7 (Cat. # 25-0038-42, clone: SK3) and 5 *μ*L of Anti-Human CD8 APC-eFluor 780 (Cat. # 47-0088-42, clone: RPA-T8) monoclonal antibody at room temperature in the dark for 20 min. The cells were next stained with FITC-conjugated anti-IFN-*γ* (Cat. #11-7319-41, clone: 4SB3), anti-human IL-4 APC (Cat. # 17-7049-41, clone: BD4-8), anti-human IL-17A PE (Cat. # 12-7178-41 clone: eBio64cAP17), anti-human IL-9 PE (Cat. # 12-7098-41, clone: MH9D1), and anti-human IL-22 eFluor 660 monoclonal antibodies (Cat. # 50-7229-41, clone: 22URTI) after fixation and permeabilization, and the doses were all 5 *μ*L. All the antibodies were purchased from eBioscience, San Diego, CA, USA. Isotype controls were given to enable correct compensation and confirm antibody specificity. Fix & Perm reagents were from Invitrogen (Carlsbad, CA, USA). All samples were washed and collected using a BD FACSCalibur Flow Cytometer. Data were analyzed with Kaluza. For analysis, we first gated CD3+CD8− lymphocytes, then we analyzed the proportion of Th1 (CD3+CD8−IFN-*γ*+ T cells), Th2 (CD3+CD8-IL4+ T cells), Th9 (CD3+CD8−IL9+ T cells), Th17 (CD3+CD8−IL−17A+ T cells), and Th22 (CD3+CD8−IL−22+ T cells).

For analysis of Treg cells, PBMCs were aliquoted into tubes and surface-labeled with 5 *μ*L of Anti-Human CD4 FITC (Cat. #11-0047-41, clone: RPA-T4) and 5 *μ*L of Anti-Human CD25 APC (Cat. #17-0259-42, clone: BC96) monoclonal antibody followed by fixation and permeabilization and then intracellular staining with 5 *μ*L of Anti-Human Foxp3 PE (Cat. # 12-4776-41, clone: RPA-T4). All the antibodies were purchased from eBioscience, San Diego, CA, USA.

### 2.6. Statistical Analysis

Continuous variables were compared with the Mann–Whitney *U* test or Kruskal-Wallis *H* test. Categorical variables were compared with the Chi-squared test. Associations between genotype were assessed by calculating odds ratios (OR) and corresponding 95% confidence intervals (CI). All computations were performed with SPSS software 22.0. GraphPad Prism 6.0 system was used to draw and analyze the survival curves. A probability (*P*) value of <0.05 was considered statistically significant.

## 3. Results

### 3.1. Sample Characteristics

SNPs of four genes in 355 patients and 350 controls were genotyped by TaqMan assays. As shown in Table 1, the gender or age of MM patients was matched with that of controls, and no significant difference was found (*P* > 0.05). Among 355 MM patients, there were 163 cases of IgG, 73 cases of IgA, 107 cases of light chain (*κ* 51 cases, *λ* 56 cases), 9 cases of IgD, 1 case of IgM, 1 case of IgE, and 1 case of nonsecretory type. The MM group was further divided into three subgroups including 11 cases at stage I, 137 at stage II, and 207 at stage III according to DS, and 50 cases at stage I, 145 at stage II, and 160 at stage III according to ISS.

### 3.2. The Relationship between NLRP3 Inflammasome Genetic Polymorphism and MM Susceptibility

All SNPs in healthy controls were consistent with the Hardy-Weinberg equilibrium. There was no significant difference in the polymorphic distribution of IL-1*β* (rs16944) (*χ*^2^ = 0.879, *P* = 0.644), IL-18 (rs1946518) (*χ*^2^ = 0.226, *P* = 0.893), CARD8 (rs2043211) (*χ*^2^ = 4.672, *P* = 0.097), and NF-*κ*B-94 ins/del ATTG (*χ*^2^ = 2.489, *P* = 0.288) between the patients and controls. The alleles of IL-1*β* (rs16944), IL-18 (rs1946518), CARD8 (rs2043211), and NF-*κ*B-94 ins/del ATTG were analyzed, respectively, and it was found that all alleles were not susceptible to MM (Table 3).

As for the genotypes, compared with the controls, the AT genotype of CARD8-C10X (rs2043211) was upregulated in MM patients and contributed to MM susceptibility (OR = 1.147, 95% CI: 1.006, 1.307 *P* = 0.039) (Table 3). The genotypes of IL-1*β* (rs16944), IL-18 (rs1946518), and NF-*κ*B-94 ins/del ATTG were not significantly different from each other.

### 3.3. The Association of Different Genotypes with Patients' Characteristics

For each SNP, patients were stratified according to their specific genotypes and these, respectively, were analyzed for the correlation of the frequencies of IL-1*β* (rs16944), IL-18 (rs1946518), CARD8 (rs2043211), and NF-*κ*B-94 ins/del ATTG with various clinical and laboratory features such as age, gender, subtype, BM myeloma cell percentage, bone damage, WBC (white blood cell), hemoglobin, platelet, and clinical stage (DS and ISS stage) in MM patients (Table 4).

We found that there was no significant difference in age, gender, MM subtypes, platelet, and bone damage between patients with the different genotypes of the above four genetic polymorphisms (*P* > 0.05). However, some genotypes were found associated with clinical stage, myeloma cell ratio, and peripheral blood hemoglobin concentration. CARD8-C10X TT patients had earlier clinical stage than CARD8-C10X AA (DS: *P*_1_ = 0.016, ISS: *P*_1_ = 0.010). CARD8-C10X AA patients also had the lowest WBC compared with CARD8-C10X TT and AT (AA<AT<TT, *P*_1_ = 0.022, *P*_2_ = 0.023). Compared with NF-*κ*B-94 ins/del ATTG ins/ins and ins/del, del/del patients had the highest myeloma cell ratio (*P*_1_ = 0.036, *P*_2_ = 0.19). Patients with IL-18 (rs16944) TT had the highest hemoglobin concentration (GG<GT<TT, *P*_1_ = 0.03, *P*_2_ = 0.249).

### 3.4. The Relationship between Genotypes of Genetic Polymorphism and Overall Survival

**Figure 1:**
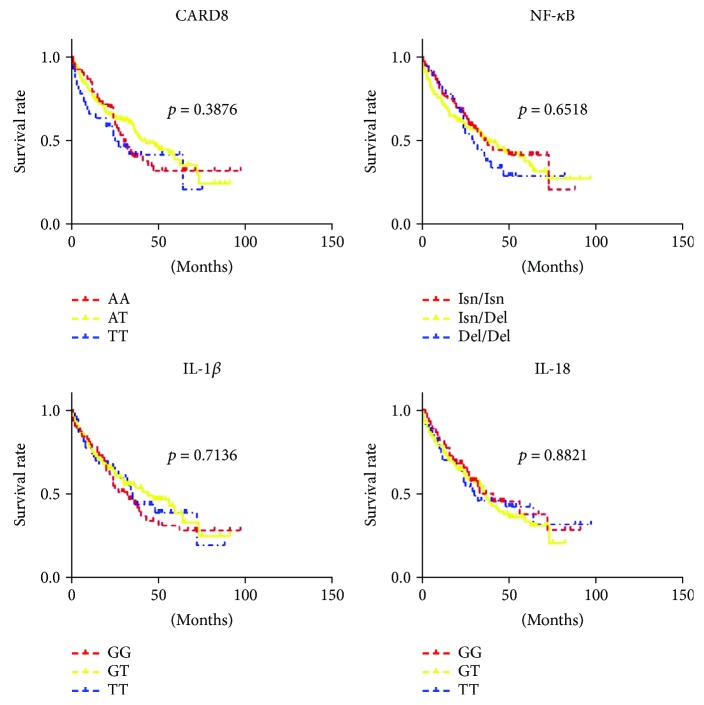
Survival curves of CARD8 (rs2043211), IL-1*β* (rs16944), IL-18 (rs1946518), and NF-*κ*B-94 ins/del ATTG genotypes in MM patients. Log-rank *P* values compared with the different genotypes are displayed, and no significant difference was found for the overall survival of MM patients with the above genotypes.

For each SNP, patients were stratified according to their specific genotypes and the survival data was described in Figure 1. No significant difference was found for the overall survival of MM patients with CARD8-C10X (rs2043211) (*P* = 0.388), IL-1*β* (rs16944) (*P* = 0.714), IL-18 (rs1946518) (*P* = 0.882), or NF-*κ*B-94 ins/del ATTG (*P* = 0.652).

### 3.5. The Expressions of NLRP3 Inflammasome-Related Genes Were Associated with Their Genetic Polymorphisms in MM Patients

**Figure 2:**
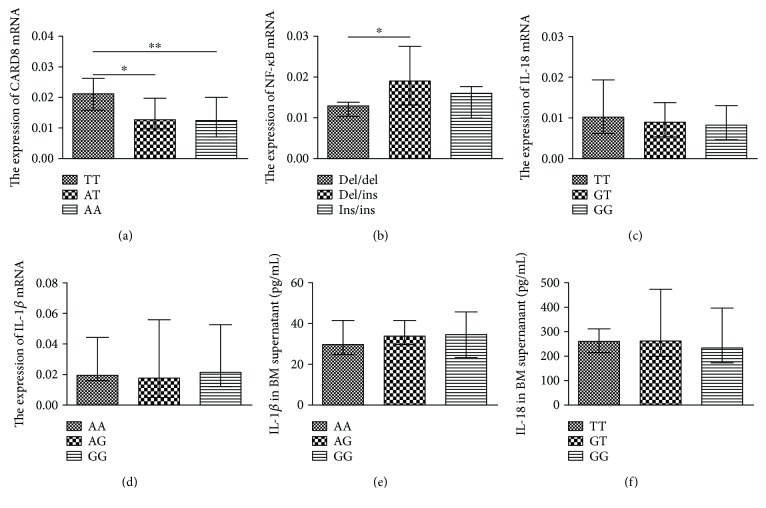
The expression of NLRP3 inflammasome-related genes was associated with the genetic polymorphisms in MM patients. (a) The relative expression of CARD8 mRNA in the genotype of the rs2043211 in MM patients. (b) The relative expression of NF-*κ*B mRNA in NF-*κ*B polymorphisms. (c-d) The association between the genetic polymorphisms of rs1946518 (c) or rs16944 (d) with their relative mRNA expression in MM patients. (e-f) IL-1*β* in the three genotypes of rs16944 (e) and IL-18 in the three genotypes of rs1946518 (f) in the BM supernatant. ^∗^*P* < 0.05 and ^∗∗^*P* < 0.01.

To investigate whether the polymorphisms of NLRP3 inflammasome-related genes were associated with the gene expression, the mRNA and cytokines in the BMMCs and supernatant were examined in 42 MM patients. MM patients with the AA or AT genotype displayed significantly decreased expression of CARD8 mRNA compared to the TT genotype of the rs2043211 of CARD8 (*P* < 0.05; Figure 2(a)). Furthermore, carriers of the ins/del genotype showed an increased expression of NF-*κ*B mRNA compared to del/del in NF-*κ*B polymorphism (*P* < 0.05; Figure 2(b)). However, no significant relationship was found between the polymorphism and expression of the other two gene polymorphisms, IL-18 rs1946518 and IL-1*β* rs16944 (Figures 2(c), 2(d)). Moreover, in the BM supernatant of MM patients, the concentration of IL-1*β* was 29.24 ± 8.93, 33.45 ± 22.11, and 34.10 ± 12.84 pg/mL in the three genotypes of IL-1*β* rs16944, AA, AG, and GG (*P* ≥ 0.05; Figure 2(e)), respectively; and in IL-18, it was 259.75 ± 155.52, 260.00 ± 5168.29, and 232.50 ± 157.96 pg/mL in the three genotypes of IL-18 rs1946518, TT, GT, and GG (*P* ≥ 0.05; Figure 2(f)), respectively.

### 3.6. The Genotypes of Gene Polymorphisms May Affect the Downstream Gene Expression

**Figure 3:**
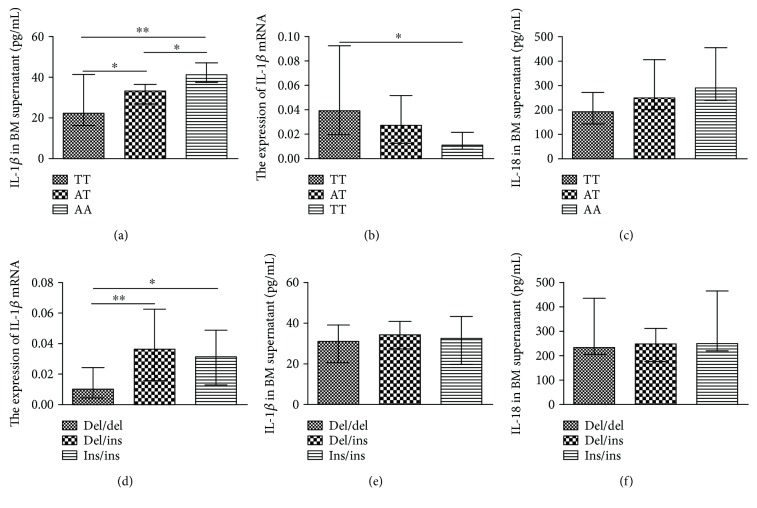
The genotypes of gene polymorphisms may affect the downstream gene expression, mRNA, or protein in MM patients. (a) The relationship between the expression of IL-1*β* in the BM supernatant and rs2043211 of CARD8. (b) The relative mRNA expression in the three genotypes of rs2043211 of CARD8. (c) The association between the expression of IL-18 in the BM supernatant and rs2043211 of CARD8. (d) The mRNA expression of IL-1*β* in the genotypes for the NF-*κ*B polymorphism. (e-f) The expression of IL-1*β* (e) and IL-18 (f) in the BM supernatant in the genotypes for the NF-*κ*B polymorphism. ^∗^*P* < 0.05 and ^∗∗^*P* < 0.01.

**Figure 4:**
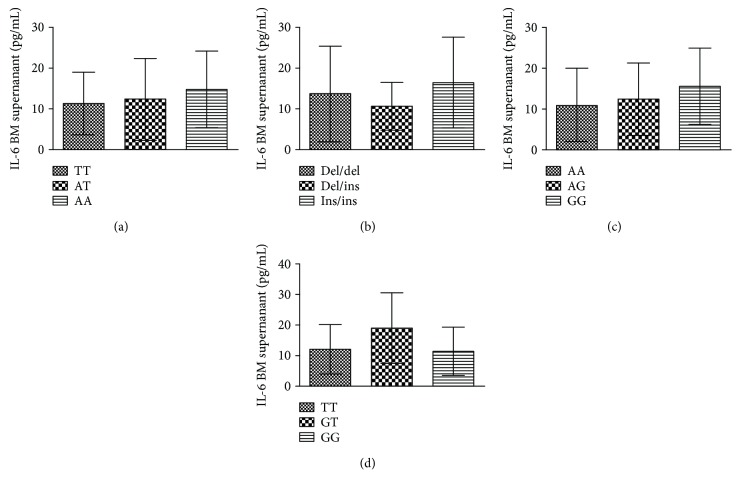
The NLRP3 gene polymorphisms may not affect the IL-6 concentration in the BM supernatant of MM patients. The relationship between the IL-6 expression in the BM supernatant with rs2043211 of CARD8 (a), NF-*κ*B-94 ins/del ATTG genotypes (b), rs16944 of IL-1*β* (c), or rs1946518 of IL-18 (d).

We analyzed the association between the four gene polymorphisms and related gene expression. We found that the IL-1*β* cytokine was 22.16 ± 8.53, 32.97 ± 8.59, and 41.41 ± 22.15 pg/mL in the TT, AT, and AA genotypes, respectively (*P* < 0.01, Figure 3(a)). However, interestingly, the mRNA expression of IL-1*β* in the order of the TT, AT, and AA genotypes showed a decreased tendency in rs2043211 (*P* < 0.05, Figure 3(b)). However, there was no association between the expression of IL-18 and rs2043211 of CARD8 (Figure 3(c)). The IL-1*β* mRNA was significantly increased in the del/del and del/ins genotypes compared to the ins/ins genotype for the NF-*κ*B polymorphism (*P* < 0.05; Figure 3(d)). However, the IL-1*β* in the BM supernatant was not associated with the NF-*κ*B polymorphism (Figure 3(e)). Furthermore, the inflammatory cytokine, IL-18, was 232.5 ± 196.964, 248.25 ± 153.521, and 249 ± 153.376 pg/mL in the NF-*κ*B polymorphism, respectively, without statistical significance (Figure 3(f)). We also determined IL-6 concentrations in BM supernatants of MM patients and analyzed their relationship with the genetic polymorphisms of CARD8-C10X (rs2043211), NF-*κ*B-94 ins/del (rs28362491), IL-1*β* (rs16944), or IL-18 (rs1946518). However, no significant difference was found between them (Figure 4).

### 3.7. The NLRP3 Genetic Polymorphisms May Be Associated with the Th Subsets in PBMCs of MM Patients

**Figure 5:**
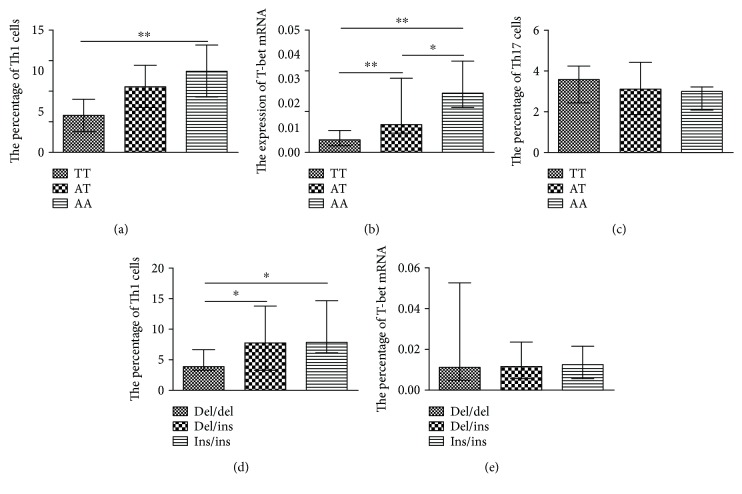
The genotypes of gene polymorphisms may be associated with the T helper subsets in PBMCs of MM patients. (a) The percentage of Th1 cells in TT, AT, and AA in rs2043211 of CARD8. (b) The relative expression of T-bet mRNA in TT, AT, and AA of rs2043211 of CARD8. (c) The percentage of Th17 in the genotypes of rs2043211. (d) The percentage of Th1 cells in del/del, del/ins, and ins/ins of NF-κB, respectively. (e) The association between the relative mRNA expression of T-bet and the genotypes of NF-*κ*B. ^∗^*P* < 0.05 and ^∗∗^*P* < 0.01.

Significant differences on Th cells and their transcription factors were observed on MM patients compared to healthy controls [11]. Therefore, we analyzed the association between the NLRP3 inflammasome-related genetic polymorphisms and Th cells (Th1, Th2, Th9, Th17, and Th22) and Treg cells in MM patients. The percentage of proinflammatory Th1 cells were 4.34 ± 2.89%, 7.57 ± 3.56%, and 9.76 ± 3.79% in TT, AT, and AA in rs2043211 of CARD8, respectively (*P* < 0.05, Figure 5(a)). In the meantime, the expression of T-bet mRNA, the key transcription factor of Th1 cells, displayed an increased tendency in the TT, AT, and AA of rs2043211 (*P* < 0.05, Figure 5(b)). The percentage of another proinflammatory Th subset, Th17, was not statistically different in the genotypes of rs2043211 (Figure 5(c)). Moreover, the percentages of Th1 cells were 3.88 ± 2.25%, 7.75 ± 5.40%, and 7.93 ± 5.48% in del/del, ins/del, and ins/ins of NF-*κ*B, respectively (Figure 5(d)). However, we did not find a significant difference between the mRNA expression of T-bet and the genotypes of NF-*κ*B (Figure 5(e)). Furthermore, the other Th cells, Th2, Th9, Th22, and Treg, had no significant differences with the gene polymorphisms in this study.

## 4. Discussion

MM is a plasma cell malignancy characterized by complex heterogeneous cytogenetic abnormalities. MM cells interact with bone marrow stromal cells (BMSCs) to stimulate the transcription and secretion of cytokines from BMSCs. Cytokines in turn not only promote MM cell growth, but also increase resistance to conventional therapies [12, 13]. In recent years, more and more studies have shown that an inflammasome in tumor microenvironments can trigger carcinogenesis, and has significant impacts on tumor immunity and immunotherapy, thus providing new insight into the effective therapies [14, 15]. The most studied inflammasome is the NLRP3 inflammasome, and mounting evidences support that the NLRP3 inflammasome plays an important role in the development of malignant tumors [16, 17]. It consists of the NLRP3 protein, ASC, and caspase-1 protein. The activated NLRP3 inflammasome, as an intracellular multimeric complex, takes part in the innate immune response against pathogens and triggers the activation of caspase-1 and the maturation of proIL-1*β* and proIL-18 [18]. It has been suggested that cytokine polymorphisms may play a role in the susceptibility to immune-mediated diseases including MM [19].

More and more researches indicate that the imbalance of the T lymphocyte subsets and cytokine network may play important roles in various tumors [20]. Th cells are the main regulators and effectors in the immune response. Several studies have revealed a critical role of Th cells in the progression of MM and proved that there are significant differences on Th subsets and their corresponding transcription factors between MM patients and healthy controls [11, 21]. However, there is no data on the association of the genetic polymorphism of the NLRP3 inflammasome with Th subsets in multiple myeloma.

Firstly, we evaluate the possible role of the NLRP3 inflammasome-related genetic polymorphisms and expressions with the susceptibility of MM. NF-*κ*B regulates the transcription of many genes for immune response, cell adhesion, differentiation, proliferation, angiogenesis, and apoptosis [22]. Abnormalities in the NF-*κ*B regulation are involved in multiple human pathologies including inflammatory diseases, immune deficiencies, diabetes, and atherosclerosis, as well as tumors [23]. Multiple kinds of NF-*κ*B pathway mutations have been found in various lymphomas, including MM [23], and Borotizolam as a protease inhibitor affects the NF-*κ*B pathway to effectively treat multiple myeloma [24]. There are several studies about the NF-*κ*B-94 ins/del polymorphism in neoplastic diseases, which showed that this polymorphism may be associated with the increased risk of epithelial ovarian cancer and oral squamous cell carcinoma [25, 26]. In our study, we found that the NF-*κ*B-94 ins/del ATTG polymorphism is not a susceptible factor for MM, but MM patients with the NF-*κ*B-94 ins/del ATTG del/del genotype had the highest myeloma cell ratio compared with ins/ins and ins/del patients. Thus, we speculated the del/del genotype may promote the proliferation of myeloma cells. We also found that the patients with the ins/del genotype showed an increased expression of NF-*κ*B compared to del/del patients. NF-*κ*B regulates the transcription of proteins that mediate cell cycle progression, apoptosis, drug resistance, and cytokine and chemokine production, thus playing a key role in the pathogenesis of MM [27]. In addition, the IL-1*β* mRNA was significantly increased in the ins/del and del/del genotype compared to the ins/ins genotype. Therefore, as a key mediator of IL-1*β*, the gene polymorphism of NF-*κ*B may influence the expression of IL-1*β*.

IL-1*β* is located on chromosome 2q14 that contains a cluster of the other IL-1 genes. It is well known that IL-1*β* is an important proinflammatory cytokine that promotes growth, invasiveness, and metastasis of a variety cancer cells [28, 29]. The IL-1*β* polymorphism and its expression play important roles in this process. IL-1*β* contains several single nucleotide polymorphisms. One of them is IL-1*β* (rs16944), which in the promoter region has been associated with increased IL-1*β* production [30] and with increased risk of developing cancers, such as gastric carcinomas and ovarian cancer [31, 32]. IL-1*β* is an osteoblast activating factor which plays an important role in the bone lesion by multiple myeloma cells, and promotes the proliferation of myeloma cells by inducing IL-6 generation [33]. Thus, IL-1*β* plays an important role in the development of multiple myeloma. However, in the present study, we found no significant difference in the distribution of the IL-1*β* (rs16944) polymorphism between MM patients and controls, and neither the G nor A allele was a susceptible factor for MM. Moreover, the IL-1*β* (rs16944) polymorphism did not affect the IL-1*β* expression and the overall survival of MM patients.

IL-18 is another important proinflammatory cytokine. Multiple studies have shown that it is a double-edged sword in the development of tumors. On one hand, it can enhance NK cell activity, induce apoptosis, and inhibit angiogenesis to exert antitumor effects [34]. On the other hand, it has tumor-promotor effects in tumor progression through inducing angiogenesis, metastasis, and immune escape [35]. Studies demonstrated that targeting IL-18 in the tumor microenvironments may improve the efficiency of cancer immunotherapy [36]. However, in our study, the genotype of IL-18 (rs1946518) was not associated with MM, and no significant difference was found for the overall survival of patients with IL-18 (rs1946518). However, hemoglobin concentration in patients with the TT genotype was significantly higher than GG and GT patients. Thus, the IL-18 (rs16944) polymorphism may contribute to the clinical manifestation of MM.

CARD8 is a new member of the caspase-associated recruitment domains (CARD) family, which are protein-protein interaction modules found extensively in proteins. Razmara et al. demonstrated that CARD8 played important roles in apoptosis and may be a negative regulator of NF-*κ*B and caspase-1 activation through the study of leukemia cell lines THP-1 and U937 [37]. The CARD8 protein is encoded by the 13 exons of the CARD8 gene on chromosome 19q13. The SNP rs2043211 of CARD8 changes cysteine at codon10 to a premature termination codon, thus yielding a premature, truncated protein, which influences the protein function [38]. So far, more and more researches proved that the CARD8 SNP was associated with the increased risk of various cancers, such as nodular melanoma, hepatocellular carcinoma, and cervical cancer [38–40]. In our study, we found that the AT genotype of CARD8-C10X (rs2043211) was associated with the increased risk of MM. Further analysis showed that CARD8-C10X TT patients had an earlier clinical stage than CARD8-C10X AA and AT patients. Thus, TT patients may have a mild condition. Furthermore, the WBC count in the three CARD8 genotypes showed a rising trend (AA<AT<TT). However, CARD8-C10X (rs2043211) did not contribute to the overall survival of MM patients. We first determined the relationship between the expression of IL-1*β* and CARD8-C10X (rs2043211), which is a negative regulation gene of the IL-1*β* cytokine. The expression of IL-1*β* had a rising trend in the TT, AT, and AA genotypes. However, the mRNA expression of IL-1*β* showed a decreased tendency in turn of the TT, AT, and AA genotypes. Thus, the relationship between IL-1*β* and CARD8-C10X (rs2043211) is complex and needs further study.

IL-6 is one of the most important and well characterized cytokines in MM. It can trigger cellular signaling, promote MM cell growth and survival, and confer drug resistance [41]. In our study, we analyzed the relationship between the different genotypes of IL-1*β* (rs16944), IL-18 (rs1946518), CARD8 (rs2043211), and NF-*κ*B-94 ins/del ATTG and the expression of IL-6 in the BM supernatant, and found that there was no significant correlation between them.

We also investigated the relationship between the genetic polymorphism of the NLRP3 inflammasome and Th cells in multiple myeloma. It is reported that Th1 and Th17 cells along with their transcription factor levels T-bet and RORC were considerably increased in MM compared with that in healthy controls [11]. Th1 cells provoke the production of IL-6 and IL-1*β* by macrophages [42], and have the effect of promoting MM progression. We found that the percentage of Th1 and its transcription factor T-bet had statistical differences among the three genotypes of CARD8-C10X (rs2043211) (TT<AT<AA). However, there is no significant difference for Th17 in the three CARD8 genotypes. Furthermore, we found that the percentage of Th1 cells was related to the NF-*κ*B-94 ins/del ATTG polymorphism and del/del patients had a lower percentage than that of ins/del and ins/ins patients. However, the T-bet mRNA expression level was not significantly different in the three genotypes of the NF-*κ*B-94 ins/del ATTG. Although accumulating evidences demonstrate that Th2, Th9, Th17, Th22, and Treg play important roles in the development of MM [21, 43, 44], we found no correlation between these Th cells and IL-1*β* (rs16944), IL-18 (rs1946518), CARD8-C10X (rs2043211), and NF-*κ*B-94 ins/del (rs28362491) polymorphisms.

In conclusion, our study indicated that the CARD8-C10X (rs2043211) AT genotype might contribute to MM susceptibility. The genetic polymorphisms of CARD8-C10X (rs2043211), NF-*κ*B-94 ins/del ATTG (rs28362491), and IL-18 (rs1946518) contribute to the clinical manifestations of multiple myeloma. Furthermore, we first investigated the relationship between the genetic polymorphism of the NLRP3 inflammasome and Th cells in MM, and found that the genotypes of CARD8-C10X (rs2043211) and NF-*κ*B-94 ins/del ATTG are closely related to Th1. Therefore, the genetic polymorphisms of the NLRP3 inflammasome associated with Th cells might be involved in the pathogenesis of multiple myeloma, which needs further study in the future.

## Figures and Tables

**Table 1:** Samples characteristics.

Parameter	Number	
	MM (*n* = 355)	Control (*n* = 350)
Male : female (ratio)	217/138 = 1.57	202/148 = 1.36
Media age (years)	60 (25–89)	55 (26–90)
*MM subtypes*		
IgG	163 (45.90%)	
IgA	73 (20.60%)	
IgM	1 (0.28%)	
IgD	9 (2.53%)	
IgE	1 (0.28%)	
*κ*	51 (14.37%)	
*λ*	56 (15.77%)	
Nonsecretory type	1 (0.28%)	
*DS stages*		
I	11 (3.10%)	
II	137 (38.59%)	
III	207 (58.31%)	
*ISS stages*		
I	50 (14.08%)	
II	145 (4.81%)	
III	160 (45.07%)	
*Laboratory features (average ± SD)*		
White blood cell (×10^9^/L)	5.62 ± 2.5	
Hemoglobin (g/L)	91.53 ± 25.12	
Platelet (×10^9^/L)	178.62 ± 86.15	
BM myeloma cell (%)	36.41 ± 23.88	

**Table 2:** Primer sequences.

Name	Forward primer	Reverse primer
IL-1*β*	5′-GCC CTA AAC AGA TGA AGT GCT C-3′	5′-GAA CCA GCA TCT TCC TCA G-3′
IL-18	5′-GCT TGA ATC TAA ATT ATC AGT C-3′	5′-GAA GAT TCA AAT TGC ATC TTA T-3′
NLRP3	5′-CAG ACT TCT GTG TGT GGG ACT GA-3′	5′-TCC TGA CAA CAT GCT GAT GTG A-3′
CARD8	5′-GAT GGA GTC GTA GGG GCC TGA G-3′	5′-CTCCCTCATCAGGGGCTTCACG-3′
AHR	5′-CAAATCCTTCCAAGCGGCATA-3′	5′-CGCTGAGCCTAAGAACTGAAAG-3′
T-bet	5′-TTGAGGTGAACGACGGAGAG-3′	5′-CCAAGGAATTGACAGTTGGGT-3′
GATA-3	5′-CGTCCTGTGCGAACTGTCA-3′	5′-GTCCCCATTGGCATTCCTCC-3′
RORC	5′-CAATGGAAGTGGTGCTGGTTAG-3′	5′-GGGAGTGGGAGAAGTCAAAGAT-3′
Foxp3	5′-GGAAAGGAGGATGGACGAACA-3′	5′-GGAAACCTCACTTCTTGGTCCC-3′
GAPDH	5′-GCTCTCTGCTCCTCCTGTTC-3′	5′-GTTGACTCCGACCTTCACCT-3′

**Table 3:** Genotype distribution of the different polymorphisms and their association with MM susceptibility.

Polymorphisms	MM *n* (%)	Controls *n* (%)	OR (95% CI)	*P* value
*IL-1β (rs16944)*				
GG	103 (29.0)	92 (26.3)	1	
AG	162 (45.6)	171 (48.9)	1.182 (0.830, 1.684)	0.355
AA	90 (25.4)	87 (24.9)	1.082 (0.720, 1.626)	0.704
Allele frequency				
G	368 (51.8)	355 (50.7)	1	
A	342 (48.2)	345 (49.3)	1.046 (0.849, 1.289)	0.675
*IL-18 (rs1946518)*				
GG	91 (25.6)	93 (28.1)	1	
GT	185 (52.1)	184 (45.8)	0.973 (0.683, 1.386)	0.88
TT	79 (22.3)	73 (26.0)	0.904 (0.588, 1.390)	0.646
Allele frequency				
G	367 (51.7)	370 (52.9)	1	
A	343 (48.3)	330 (47.1)	0.954 (0.774, 1.176)	0.661
*CARD8-C10X (rs2043211)*				
AA	89 (25.1)	108 (30.9)	0.69 (0.485, 0.982)	
AT	184 (51.8)	154 (44.0)	1.147 (1.006, 1.307)	0.039
TT	82 (23.1)	88 (25.1)	0.884 (0.586, 1.334)	0.558
Allele frequency				
A	362 (51.0)	370 (52.9)	1	
T	348 (49.0)	330 (47.1)	0.928 (0.753, 1.143)	0.482
*NF-κB-94 ins/del (rs28362491)*				
Ins/ins	130 (36.6)	109 (31.1)	1	
Ins/del	160 (45.1)	175 (50)	1.304 (0.935, 1.820)	0.117
Del/del	65 (18.3)	66 (18.9)	1.211 (0.79, 1.856)	0.379
Allele frequency				
Ins	420 (59.2)	393 (56.1)	1	
Del	290 (40.8)	307 (43.9)	1.131 (0.916, 1.398)	0.252

**Table 4:** The correlations of the genetic polymorphisms of the NLRP3 inflammasome with clinical features of MM patients.

	Age	Male : female	Type							DS			ISS			Bone damaged		Hemogram			BM myeloma
			IgG	IgA	Light chain (*κ* + *λ*)	IgD	IgE	IgM	Nonsecretory type	I	II	III	I	II	III	With	Without	WBC (×10^9^/L)	Hemoglobin (g/L)	Platelet (×10^9^/L)	Cell percentage (%)
*CARD8-C10X (rs2043211)*																					
AA	59 (25–80)	55 : 34	44	14	30	1	0	0	0	3	29	57	12	43	34	72	17	5.20 ± 0.28	88.95 ± 2.52	177.96 ± 8.96	34.04 ± 2.40
AT	59 (28–89)	107 : 77	78	39	59	5	1	1	1	8	73	35	25	79	80	153	31	5.71 ± 0.18	92.11 ± 1.87	183.97 ± 6.74	36.47 ± 1.78
TT	61 (25–84)	55 : 27	41	20	18	3	0	0	0	10	35	37	23	23	36	70	12	5.87 ± 0.28	93.03 ± 2.89	167.34 ± 8.26	36.99 ± 2.63
*P*_1_	0.175	0.524		0.18						0.016			0.01			0.437		0.022	0.331	0.598	0.41
*P*_2_	0.608	0.109		0.512						0.446			0.647			0.647		0.023	0.324	0.758	0.475
*NF-κB-94 ind/del (rs28362491)*																					
Ins/ins	60 (25–89)	77 : 53	61	28	36	3	0	1	0	8	48	74	25	51	54	103	27	6.7 ± 0.10	100.4 ± 0.23	219.61 ± 0.54	49.99 ± 0.37
Ins/del	60 (25–80)	98 : 62	69	37	48	6	1	0	0	2	65	93	18	61	81	135	25	6.76 ± 0.10	97.57 ± 0.21	222.37 ± 0.59	51.39 ± 0.30
Ins/del	58 (29–77)	42 : 23	33	9	23	0	0	0	0	1	24	40	7	33	25	57	8	6.69 ± 0.19	96.32 ± 0.33	215.13 ± 0.88	54.36 ± 0.40
*P*_1_	0.306	0.767		0.059						0.155			0.096			0.28		0.062	0.266	0.561	0.036
*P*_2_	0.352																	0.38	0.324	0.601	0.19
*IL-1β (rs16944)*																					
GG	60 (28–89)	66 : 37	46	18	35	4	0	0	0	3	36	64	14	39	50	88	15	6.77 ± 0.12	95.80 ± 0.26	220.61 ± 0.70	52.1 ± 0.39
AG	60 (25–84)		76	35	47	3	1	1	0	6	72	84	24	72	66	131	31	6.52 ± 0.09	101.35 ± 0.21	224.19 ± 0.52	50.55 ± 0.31
AA	59 (35–79)		41	21	25	2	0	0	0	4	29	57	12	34	44	76	14	7.04 ± 0.15	95.80 ± 0.25	211.43 ± 0.77	52.37 ± 0.36
*P*_1_	0.338	0.317		0.84						0.303			0.681			0.579		0.462	0.738	0.503	0.525
*P*_2_	0.995																	0.245	0.156	0.588	0.322
*IL-18 (rs1946518)*																					
GG	58 (29–91)	58 : 33	43	15	31	1	0	0	0	2	34	55	11	37	43	79	12	6.87 ± 0.13	95.12 ± 0.29	211.40 ± 0.38	52.62 ± 0.38
GT	60 (28–89)	107 : 78	85	37	55	7	1	1	0	7	68	110	28	73	84	154	31	6.57 ± 0.09	97.83 ± 0.19	218.92 ± 0.52	51.17 ± 0.28
TT	61.5 (25–79)	52 : 27	35	22	21	1	0	0	0	4	35	40	11	35	33	62	17	6.94 ± 0.15	103.15 ± 0.29	232.36 ± 0.85	51.12 ± 0.45
*P*_1_	0.399	0.399		0.422						0.604			0.908			0.351		0.395	0.03	0.951	0.496
*P*_2_	0.168																	0.429	0.249	0.839	0.987

*P*_1_, *P* values of homozygous polymorphisms. *P*_2_, *P* values of heterozygous polymorphisms.

## Data Availability

The data used to support the findings of this study are available from the corresponding author upon request.
